# The longitudinal relationship between executive dysfunction and reactive and proactive aggression in adolescents: impulsivity as a mediator and sex differences

**DOI:** 10.3389/fpsyt.2024.1484340

**Published:** 2024-12-20

**Authors:** Xiaojie Su, Wenjie Li, Changsheng Hu, Huimin Liu, Rong Lian

**Affiliations:** ^1^ School of Psychology, Fujian Normal University, Fuzhou, China; ^2^ Normal School, Urumqi Vocational University, Urumqi, China; ^3^ School of Teacher Education, Sichuan Vocational and Technical College, Suining, China; ^4^ School of Psychology, Hainan Normal University, Haikou, China

**Keywords:** adolescents, impulsivity, reactive aggression, proactive aggression, executive dysfunction

## Abstract

**Background:**

Adolescent aggression poses a significant threat to mental health and social functioning. This study investigated the underlying mechanisms of reactive and proactive aggression in Chinese adolescents, focusing on the roles of executive dysfunction, impulsivity, and sex. We hypothesized that executive dysfunction would predict both reactive and proactive aggression, with impulsivity mediating these relationships. Furthermore, we explored the moderating role of sex in the association between impulsivity and both types of aggression.

**Method:**

A longitudinal design with a 1-year follow-up was employed. The sample comprised 617 middle school students (mean age = 15.26 years at Time 2; 59% male). Participants completed self-report questionnaires at two time points (T1: April 2023; T2: April 2024): the Reactive-Proactive Aggression Questionnaire (RPQ), the Teenage Executive Functioning Inventory (TEXI), and the Barratt Impulsiveness Scale (BIS).

**Results:**

Executive dysfunction at T1 significantly predicted both proactive and reactive aggression at T2. Impulsivity at T2 partially mediated the relationship between executive dysfunction at T1 and both proactive and reactive aggression at T2. Sex moderated the relationship between impulsivity and aggression. Specifically, impulsivity at T2 significantly predicted proactive aggression at T2 only in male adolescents. Impulsivity at T2 significantly predicted reactive aggression at T2 in both male and female adolescents, with a stronger effect observed in males.

**Conclusion:**

Our findings suggest that executive dysfunction contributes to adolescent aggression both directly and indirectly through increased impulsivity. Sex plays a moderating role, with male adolescents demonstrating greater vulnerability to the influence of impulsivity on aggression. These results underscore the importance of considering executive function, impulsivity, and sex in the development of interventions to prevent and reduce adolescent aggression.

## 1 Introduction

Adolescent aggression is a pressing social issue that is both prevalent and profoundly impacts the individuals involved (1). A survey of approximately 80,000 adolescents revealed that 18% reported engaging in aggressive behavior (2). For adolescents who perpetrate aggression, these experiences can lead to a cascade of negative consequences, such as substance abuse and depressive tendencies (3). Fite et al. (4) found that adolescents with a history of aggression often exhibit greater levels of anxiety and more antisocial behavior in adulthood. Meanwhile, adolescents who are victimized by aggression may experience anxiety, depression, and other psychological problems; develop fears of interpersonal relationships and trust issues; and may even suffer from long-term psychological trauma. Such psychological issues not only impact the academic performance and social functioning of these individuals but may also persist into adulthood, leading to mental health problems and social difficulties (5). Adolescence, particularly secondary school period, is a critical period for the development and expression of aggressive behavior (6). Chen et al. (7) analyzed the changes in aggression patterns that occur in the transition from eighth to ninth grade and found that the proportion of low-aggression adolescents who become high-aggression adolescents was as high as 28.8%, highlighting the importance of early identification and intervention.

Based on the functions of aggression and the emotional involvement of the aggressor, aggressive behavior can be categorized as either reactive or proactive aggression (8). Reactive aggression is impulsive, triggered by negative emotions such as anger or frustration, and serves as emotional release. It often stems from cognitive biases, where individuals with limited cognitive skills may be prone to hostile attribution errors, perceiving threats and reacting aggressively (9). This type of aggression is prevalent in emotionally strained environments, such as in families with poor communication or high parental criticism, where adolescents express distress through reactive aggression (10, 11). In contrast, proactive aggression is planned, unemotional, and goal-driven, typically occurring in contexts of power struggles or competition, such as schools where individuals seek social status or material rewards (12, 13). Overall, reactive aggression is common in emotionally charged situations, while proactive aggression is more likely to arise in strategic, competitive environments.

Given the differences in behavioral responses and cognitive processes between reactive and proactive aggression, this study, focusing on Chinese adolescents, sought to examine the underlying mechanisms of both types of aggression.

### 1.1 Executive dysfunction and aggressive behavior

Adolescence is a peak period for the onset of aggressive behavior. During this developmental stage, individuals’ executive functions, which represent a set of cognitive skills that involve top-down control processes elicited in the planning, organizing, and monitoring of complex, goal-directed behavior (14), may lag behind the demands of their environment, making it difficult for them to accurately perceive events and regulate intense aggressive emotions (15). A longitudinal study by Maloney et al. (16) found that adolescents with executive dysfunction exhibited more aggressive behavior in adulthood. Individuals with lower levels of executive function (cognitive ability) are more likely to interpret others’ intentions as hostile in ambiguous situations (17), and they tend to develop hostile attribution biases during the stages of cue encoding and interpretation, which can lead to aggressive behavior in the final stage of behavioral execution (9). Additionally, Wood and Worthington (18) suggested that damage to brain regions associated with executive function, such as the dorsolateral prefrontal cortex, orbitofrontal cortex, and ventromedial prefrontal cortex, can impair individuals’ ability to regulate emotions, make decisions, and control impulses. They also noted that executive function primarily develops during adolescence, which means that any damage during this critical period can have significant long-term effects on these abilities (18). Therefore, the level of executive function is not only related to cognitive processes but also closely linked to emotion regulation and behavioral control. These factors together may lead to individuals’ misinterpretation of social cues and intensified emotional responses, ultimately manifesting as aggressive behavior.

Previous studies have often examined aggressive behavior as a whole, with fewer studies differentiating between different types of aggressive behavior and looking at their relationship with executive dysfunction. In fact, there is evidence suggesting that the impact of executive dysfunction on different subtypes of aggression may vary (19). Proactive aggression is a premeditated, goal-directed behavior, typically undertaken to achieve a specific purpose. Executive function impairments may affect individuals’ ability to plan, organize, and make decisions. Damage to the dorsolateral prefrontal cortex may impair individuals’ “cold” executive functions, including logical reasoning and problem-solving (20), which may affect a person’s ability to plan their aggressive actions to solve problems. Reactive aggression, on the other hand, is an immediate response to social threats, provocations, or frustrations, and it is often accompanied by strong emotional reactions. Impairments in “hot” executive functions may affect individuals’ ability to regulate emotions; for example, damage to the ventromedial prefrontal cortex and orbitofrontal cortex may impair individuals’ processing of emotionally salient information, including empathy and social judgment, which may lead to hypersensitivity to provocation and an inability to appropriately regulate emotional responses (21).

Thomson and Centifanti (22) measured individuals’ executive functions and found that performance on executive function tasks (such as the Stroop task) significantly predicted both proactive and reactive aggression, with a stronger association between reactive aggression and executive function. These conclusions were verified in a 3-year longitudinal study by Rohlf et al. (23), who found that early childhood executive dysfunction positively predicted later childhood proactive and reactive aggression and that the correlation between executive dysfunction and reactive aggression was stronger than that involving proactive aggression. Based on existing theories and empirical research, the current study proposes the following two hypotheses: (1a) executive dysfunction significantly positively predicts proactive aggression and (1b) executive dysfunction significantly positively predicts reactive aggression, with a stronger correlation between adolescents’ executive dysfunction and reactive aggression.

### 1.2 The mediating role of impulsivity

Impulsivity is a broad construct that has multiple operationalizations. Definitions include risk-taking, sensation-seeking, behavioral disinhibition, preference for small immediate rewards over large distal rewards, deficits in planning, and urgency (24). This trait is particularly prevalent among adolescents, who often respond to internal and external stimuli in a hasty and unplanned manner (25). Recent research has shed light on the intricate relationship between executive function, impulsivity, and aggressive behavior. Finkel and Hall (26) proposed the I^3^ model of aggression, suggesting that aggressive behavior arises from the interaction of personality traits like impulsivity, self-regulatory capacities such as executive function, and external environmental factors. Impairments in executive function can lead to deficits in cognitive processes involved in evaluating the consequences of behavior, increasing impulsivity—the tendency to act without fully considering potential negative outcomes (27).

This relationship has also been empirically supported in behavioral experiments. Reynolds et al. (28) measured participants’ executive function using the Wisconsin Card Sorting Test and assessed their aggressive behavior and impulsivity using questionnaires. They found that participants’ executive function was negatively correlated with impulsivity and that participants’ impulsivity positively predicted their own aggressive behavior. Notably, impulsivity’s influence on aggression may vary depending on the subtype of aggression. Research indicates that impulsivity primarily predicts reactive aggression rather than proactive aggression (29), a conclusion echoed by Duan et al. (30), who found that trait impulsivity was significantly related to reactive aggression in a competitive reaction time task. Curtis et al. (31) further explored this relationship, identifying a significant conceptual and cognitive overlap between proactive and reactive aggression, suggesting that the distinction between the two types may not be as pronounced in the context of impulsivity.

Given the controversy surrounding the relationship between impulsivity and subtypes of aggression and the fact that previous cross-sectional studies could not reveal causal relationships, the present study examines the mediating role of impulsivity in the relationship between executive dysfunction and aggressive behavior using a longitudinal design, while differentiating between proactive and reactive aggression. Based on this, the present study proposes the following two additional hypotheses: (2a) impulsivity mediates the effect of executive dysfunction on proactive aggression, (2b) impulsivity also mediates the relationship between executive dysfunction and reactive aggression, and (2c) the mediating effect of impulsivity is more pronounced in the context of reactive aggression compared to proactive aggression.

### 1.3 The moderating role of sex

When discussing the relationship between impulsivity and aggressive behavior, sex is an important factor to consider. Previous research has shown that sex is closely related to aggressive behavior, with males generally exhibiting higher levels of aggression compared to females (32), especially in impulsive aggression (33). This difference can be partly explained by physiological factors, including neurotransmitters such as serotonin, which is crucial for emotional regulation and impulse control (34). Males have been found to have lower serotonin regulation abilities when faced with stress or provocation, leading to weaker impulse control and greater difficulty managing aggression (35). Additionally, testosterone, the primary male sex hormone, has been linked to increased aggression and dominance behavior, especially in competitive or resource-driven situations (36). Testosterone’s effects on brain regions involved in emotion and impulse regulation, such as the prefrontal cortex and amygdala, may further explain why males are more prone to impulsive aggression in response to provocation (36, 37).

While both executive dysfunction and impulsivity contribute to aggression, the moderating role of sex is more relevant to impulsivity than to executive function. This is because physiological factors, such as serotonin and testosterone levels, differ between males and females and directly affect impulsive behavior and aggression. In contrast, executive dysfunction is linked to cognitive control processes, which are less influenced by these biological differences (38). Therefore, when examining aggression, it is more important to consider how sex influences impulsivity, as males’ biological makeup makes them more prone to impulsive aggression.

Building on this, Dinić and Wertag (39) explored the relationship between different subtypes of aggression and sex, finding that males scored significantly higher than females in both proactive and reactive aggression. Specifically, males were more likely to exhibit reactive aggression under the same level of anger (40). The connection between impulsivity and reactive aggression is particularly strong in males, as their relatively weaker impulse control makes it more difficult to suppress aggressive responses when provoked or stressed (41). This is especially evident among male juvenile delinquents (42). In contrast, females’ aggressive behavior tends to be shaped more by social and environmental factors, such as early trauma or neglect, which are more strongly associated with proactive aggression in females (43, 44). Additionally, impulsivity in females has been linked to mental health problems, including suicidal behavior, underscoring the importance of early intervention in this population (45).

**Figure 1:**
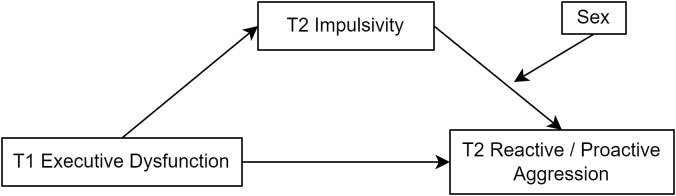
Proposed model of executive dysfunction on proactive–reactive aggression.

In summary, physiological differences in impulse control, as well as psychological factors like early experiences and communication skills, work together to influence the expression of aggressive behavior. Males may be more inclined to react immediately to external stimuli, while females may be more likely to transform internal emotions into proactive aggression. These findings collectively emphasize the importance of considering sex differences and related psychosocial factors in the prevention and intervention of adolescent aggressive behavior. Based on these findings, this study proposes hypothesis 3a: sex moderates the effect of impulsivity on proactive aggression. This study also proposes hypothesis 3b: sex moderates the effect of impulsivity on reactive aggression, as detailed in the proposed model in **Figure 1**.

## 2 Materials and methods

### 2.1 Participants

This study employed a cluster sampling method, targeting middle school students from Sichuan Province, China. A total of 630 students participated, and the final sample comprised 617 valid questionnaires, with a response rate of 97.93%. The average age of the participants was 15.26 years (SD = 1.37), with an age range of 13 to 17 years. Among the participants, 253 were female (41.0%) and 364 were male (59.0%).

### 2.2 Measure

#### 2.2.1 Reactive–proactive aggression questionnaire

The Reactive–Proactive Aggression Questionnaire developed by Raine et al. (46) was used to measure participants’ reactive and proactive aggression. This questionnaire consists of 25 items, of which 13 items measure reactive aggression (e.g., “Yelled at others when they have annoyed you”) and 12 items measure proactive aggression (e.g., “Hurt others to win a game”). The questionnaire used a 3-point Likert scale, with 0 points representing “never,” 1 point representing “sometimes,” and 2 points representing “often.” The total score of the scale was calculated, with higher scores indicating greater levels of reactive or proactive aggression. This study used the Chinese version revised by You et al. (47). In this study, the internal consistency was assessed using Cronbach’ s α. At T1, Cronbach’s α was 0.756 for reactive aggression and 0.651 for proactive aggression. At T2, these values increased to 0.807 and 0.734, respectively, indicating satisfactory to good internal consistency for both reactive and proactive aggression scales at both time points.

#### 2.2.2 Teenage executive functioning inventory

The Teenage Executive Functioning Inventory, developed by Thorell et al., was used to assess adolescents' deficits in working memory and inhibitory control (48). The questionnaire consists of 20 items, of which 11 items measure working memory (e.g., “Has difficulties with tasks involving several steps that need to be completed in a certain order”) and nine items measure inhibition (e.g., “ Puts things off until the last minute”). The questionnaire uses a 5-point Likert scale, with scores ranging from 1 point (“absolutely not”) to 5 points (“absolutely yes”). The total score of the scale was calculated, with higher scores indicating more severe executive dysfunction. The scale has good reliability and validity (48). This study used the Chinese version revised by Hu et al. (49). In this study, the internal consistency coefficients of the scale at T1 and T2 were 0.891 and 0.898, respectively.

#### 2.2.3 Barratt impulsiveness scale

The Barratt Impulsiveness Scale (50) was used to assess trait impulsivity. This questionnaire consists of 30 items and uses a 5-point Likert scale for responses, encompassing three dimensions: motor impulsivity, cognitive impulsivity, and non-planning impulsivity. Each dimension contains 10 items, such as “I do things without thinking” for motor impulsivity, “I make-up my mind quickly” for cognitive impulsivity, and “I plan tasks carefully” for non-planning impulsivity. The total score of the questionnaire was calculated for each participant, with higher scores indicating greater levels of impulsivity. This study used the Chinese version revised by Li et al. (51). In this study, the internal consistency coefficients of the scale at T1 and T2 were 0.878 and 0.888, respectively.

### 2.3 Research procedures and statistical analysis

Data collection was carried out at two time points: April 2023 (T1) and April 2024 (T2). The questionnaires were administered by trained psychology teachers and group activity organizers and were collected on-site by the assessors. Prior to the study, informed consent was obtained from all participants and their guardians. Each participant and guardian received a comprehensive description of the study procedures, including the questionnaire and any associated precautions. This information was provided by the principal investigator to ensure that all parties were fully informed before participation. This study has been approved by the Ethics Committee of Sichuan Vocational and Technical College (ethics approval number: XLJKJY2437B-1), in accordance with the Declaration of Helsinki.

A longitudinal research design was adopted to delve into the association between executive dysfunction and aggressive behavior in adolescents, as well as to examine the mediating role of impulsivity and the moderating role of sex in this relationship. We chose a longitudinal study design for two reasons: first, adolescence is a critical period for the development of aggressive behavior, and there is substantial evidence of significant shifts in adolescent aggression during this period (6, 52), highlighting the need for longitudinal studies to capture behavioral changes over time; second, longitudinal research can reveal temporal sequences among variables, providing more rigorous evidence for causal inferences.

Descriptive statistics and correlation analyses were conducted using SPSS version 26.0 (IBM Corporation, Armonk, NY, USA). To examine the mediating role of impulsivity, we applied Model 4 of the PROCESS macro (53). This model assesses whether impulsivity mediates the relationship between executive dysfunction and aggression. Additionally, to explore the potential moderating effect of sex on the mediating relationship, we utilized Model 14 of the PROCESS macro. This model allowed us to assess whether the mediating effect of impulsivity on the relationship between executive dysfunction and aggression varies by sex. All mediation assumptions were met, with significant correlations supporting the validity of the analyses.

## 3 Results

### 3.1 Common method bias

To address potential common method bias, Harman’s single-factor test was conducted on the self-reported data. The results showed that, at T1, there were 18 factors with eigenvalues greater than 1, and the first factor explained 17.52% of the total variance, which was below the critical value of 40%. At T2, there were 16 factors with eigenvalues greater than 1, and the first factor explained 19.06% of the total variance, thus also being below the critical value of 40%. Therefore, the data in this study did not have a serious common method bias problem (54).

### 3.2 Descriptive and correlation analysis

**Table 1** presents the means of the variables involved in this study. The results showed that there were no significant differences between the two measurements for all variables except proactive aggression. Overall, there were no significant differences between the two measurements. Subsequently, correlation analyses were conducted on executive dysfunction, impulsivity, and reactive and proactive aggression at T1 and T2, and the results showed that there were significant positive correlations between all pairs of variables, as seen in **Table 1**.

**Table 1:** Descriptive statistics and correlation coefficients for all variables.

Variables	M	SD	1	2	3	4	5	6	7
1.T1 Executive Dysfunction	56.97	14.24	1						
2.T2 Executive Dysfunction	57.90	13.68	0.50^***^	1					
3.T1 Impulsivity	81.72	15.26	0.58^***^	0.39^***^	1				
4.T2 Impulsivity	82.13	14.41	0.41^***^	0.57^***^	0.57^***^	1			
5.T1 Reactive Aggression	6.35	3.63	0.46^***^	0.29^***^	0.35^***^	0.31^***^	1		
6.T2 Reactive Aggression	6.60	4.12	0.34^***^	0.45^***^	0.29^***^	0.39^***^	0.45^***^	1	
7.T1 Proactive Aggression	0.86	1.40	0.16^***^	0.12^***^	0.23^***^	0.17^***^	0.39^***^	0.14^***^	1
8.T2 Proactive Aggression	1.15	1.86	0.22^***^	0.26^***^	0.24^***^	0.26^***^	0.23^***^	0.50^***^	0.28^***^

**p < 0.01, ***p < 0.001.

### 3.3 Tests of mediating effect of executive dysfunction

**Figure 2:**
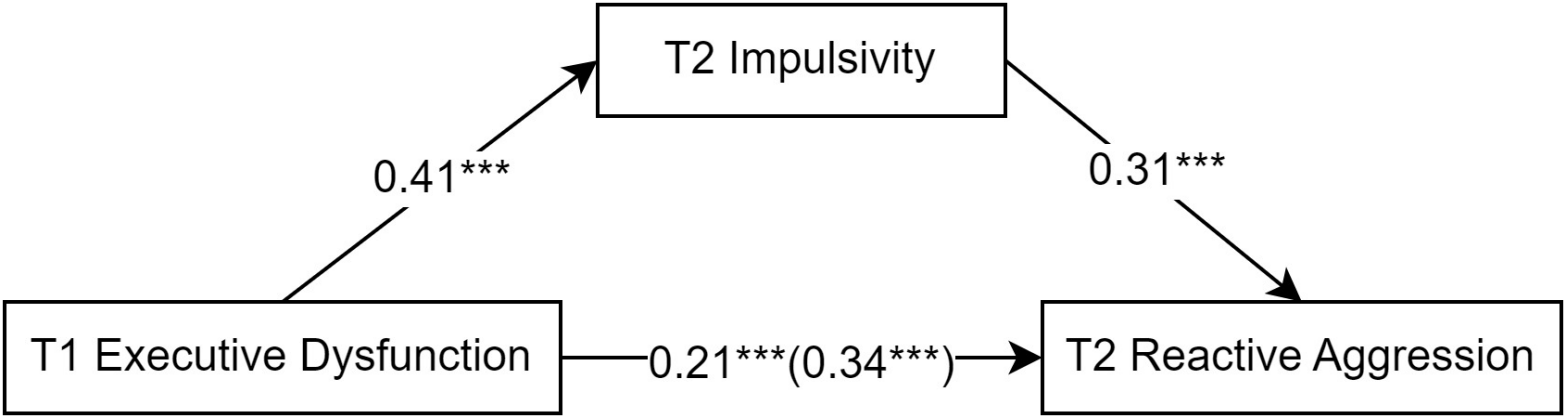
The mediating role of impulsivity in the prediction of reactive aggression by executive dysfunction. The variables in this figure have been standardized. The symbol "***" represents statistical significance, indicating ***p < 0.001.

First, Model 4 of Hayes (53) PROCESS macro was used to examine the mediating effect of impulsivity on reactive aggression (**Figure 2**). Regression analysis was conducted with T1 executive dysfunction as the independent variable and T2 reactive aggression as the dependent variable. The results showed that T1 executive dysfunction significantly and positively predicted T2 reactive aggression (*β* = 0.34, *t* = 9.02, *p* < 0.001). After adding the mediating variable T2 impulsivity, the direct effect of T1 executive dysfunction on T2 reactive aggression remained significant (*β* = 0.21, *t* = 5.38, *p* < 0.001), while the predictive effect of T1 executive dysfunction on T2 impulsivity was significant (*β* = 0.41, *t* = 11.32, *p* < 0.001) and the predictive effect of T2 impulsivity on T2 reactive aggression was significant (*β* = 0.31, *t* = 7.71, *p* < 0.001), respectively. The 95% confidence interval (CI) of the mediating effect of T2 impulsivity between T1 executive dysfunction and T2 reactive aggression was 0.08–0.17 (Sobel test = 6.38 SE = 0.02, p < 0.01), indicating a significant partial mediating effect. The mediating effect accounted for 38% of the total effect (total effect = 0.34, direct effect = 0.21).

**Figure 3:**
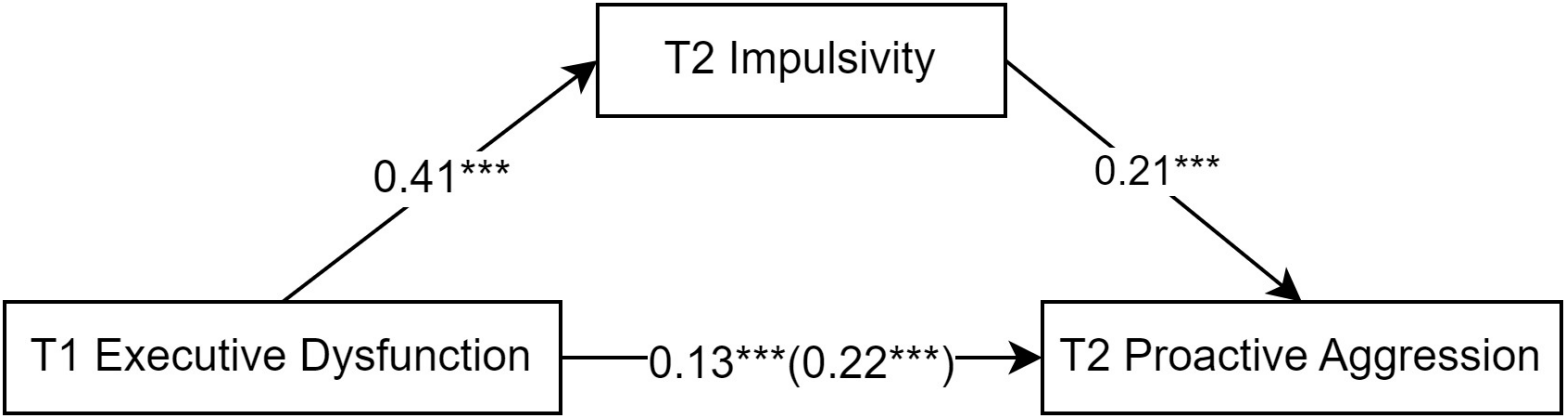
The mediating role of impulsivity in the prediction of proactive aggression by executive dysfunction. The symbol "***" represents statistical significance, indicating ***p < 0.001.

Similarly, the mediating effect of impulsivity on proactive aggression was examined (**Figure 3**). Regression analysis was conducted with T1 executive dysfunction as the independent variable and T2 proactive aggression as the dependent variable. The results showed that T1 executive dysfunction significantly and positively predicted T2 proactive aggression (*β* = 0.22, *t* = 5.58, *p* < 0.001). After adding the mediating variable T2 impulsivity, the direct effect of T1 executive dysfunction on T2 proactive aggression remained significant (*β* = 0.13, *t* = 3.11, *p* < 0.01); also, the predictive effect of T1 executive dysfunction on T2 impulsivity was significant (*β* = 0.41, *t* = 11.27, *p* < 0.001) and the predictive effect of T2 impulsivity on T2 proactive aggression was significant (*β* = 0.21, *t* = 4.99, *p* < 0.001). The 95% CI of the mediating effect of T2 impulsivity between T1 executive dysfunction and T2 proactive aggression was 0.04–0.12 (Sobel test = 4.90 SE = 0.01, p < 0.01), indicating a significant partial mediating effect. The mediating effect accounted for 36% of the total effect (total effect = 0.22, direct effect = 0.13).

### 3.4 Test of moderated mediation model effect

**Figure 4:**
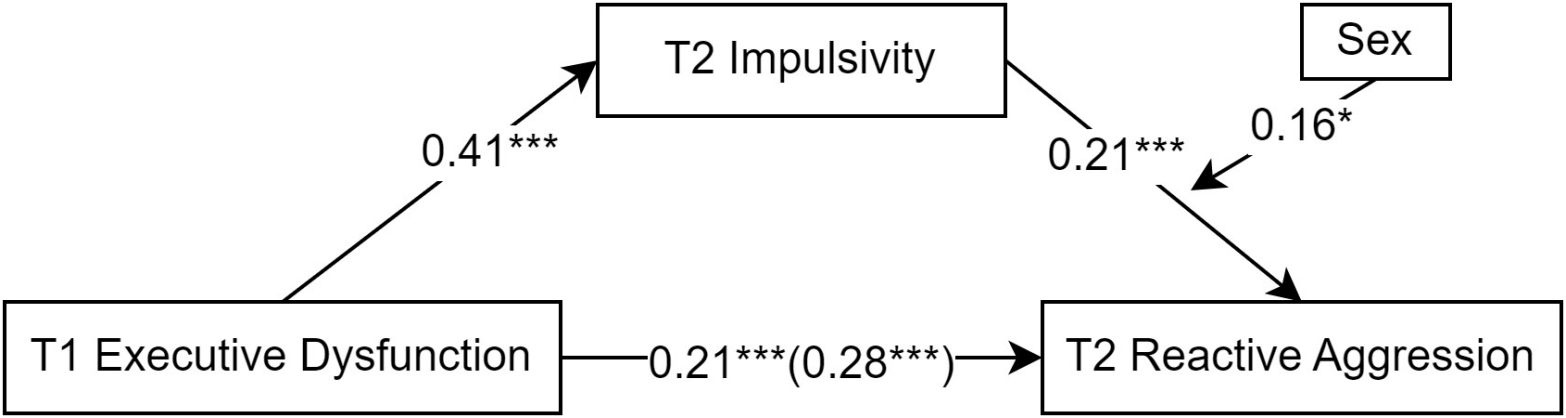
Moderated mediation model diagram of the impact of executive dysfunction on reactive aggression. *p < 0.05; ***p < 0.001.

Model 14 of Hayes (53) PROCESS macro was used to analyze the moderated mediation model with T2 reactive aggression as the dependent variable. The results showed (**Table 2**, **Figure 4**) that sex moderated the latter half of the mediation path (*β* = 0.16, SE = 0.07, *p* = 0.025, 95% CI [0.02–0.31]) (total effect = 0.28, direct effect = 0.21). The mediating effect value for the male group was 0.15, while that for the female group was 0.09.

**Table 2:** Examination of a moderated mediation model of executive dysfunction on reactive aggression.

Variables	Mediator Variable: T2 Impulsivity			Dependent Variable: T2 Reactive Aggression		
	β	SE	t	β	SE	t
T1 Executive Dysfunction	0.41^***^	0.03	11.34	0.21^***^	0.03	5.41
T2 Impulsivity				0.21^***^	0.05	3.81
Sex				0.08	0.07	1.11
T2 Impulsivity*Sex				0.16^*^	0.07	2.23
R^2^	0.17			0.20		
F	128.62			39.08		

Sex: 0 = female, 1 = male, *p < 0.05, ***p < 0.001.

**Figure 5:**
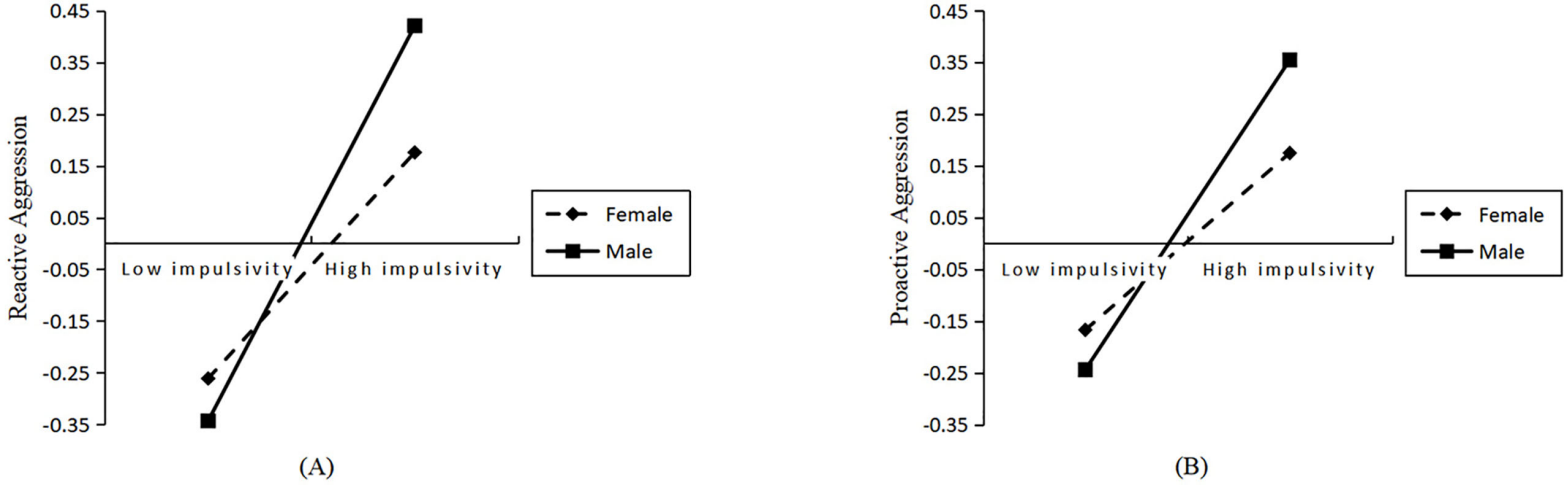
**(A)** Simple slope plot of impulsivity on reactive aggression; **(B)** Simple slope plot of impulsivity on proactive aggression. The horizontal axis represents impulsivity, and the vertical axis represents reactive or proactive aggressive behavior. The dashed line represents females, and the solid line represents males.

Further simple slope analysis showed (**Figure 5**) that T2 impulsivity had a significant positive predictive effect on T2 reactive aggression in the male group (*β* = 0.38, *p* < 0.001), while T2 impulsivity in the female group had a significant but smaller positive predictive effect on T2 reactive aggression (*β* = 0.21, *p* < 0.001).

**Figure 6:**
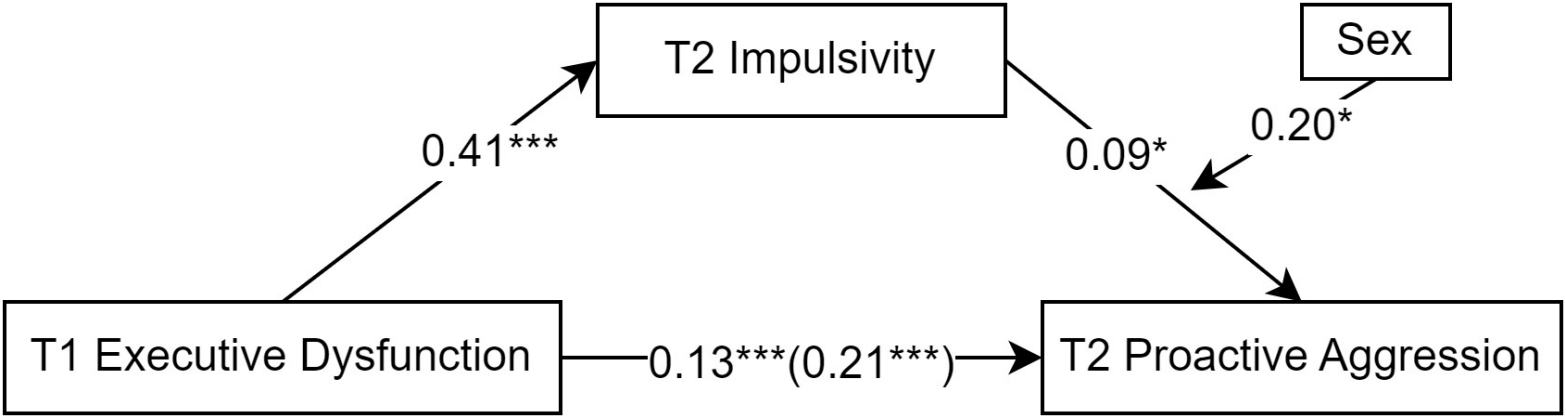
Moderated mediation model diagram of the impact of executive dysfunction on proactive aggression. *p < 0.05; ***p < 0.001.

Similarly, with T2 proactive aggression as the dependent variable, Model 14 of Hayes (53) PROCESS macro was used to analyze the moderated mediation model. The results showed (**Table 3**, **Figure 6**) that sex moderated the latter half of the mediation path (*β* = 0.20, SE = 0.07, *p* = 0.007, 95% CI [0.05–0.36]) (total effect = 0.21, direct effect = 0.13). The mediating effect value for the male group was 0.12, while that for the female group was not significant at 0.03 (p > 0.05).

**Table 3:** Examination of a moderated mediation model of executive dysfunction on proactive aggression.

Variables	Mediator Variable: T2 Impulsivity			Dependent Variable: T2 Proactive Aggression		
	β	SE	t	β	SE	t
T1 Executive Dysfunction	0.41^***^	0.03	11.29	0.13^***^	0.04	3.23
T2 Impulsivity				0.09	0.06	1.51
Sex				0.13	0.07	1.66
T2 Impulsivity*Sex				0.20^**^	0.07	2.66
R^2^	0.17			0.09		
F	127.60^***^			16.69^***^		

*p < 0.05, **p < 0.01, ***p < 0.001.

Further simple slope analysis showed (**Figure 5**) that T2 impulsivity had a significant positive predictive effect on T2 proactive aggression in the male group (*β* = 0.30, *p* < 0.001), while T2 impulsivity in the female group did not have a significant positive predictive effect on T2 proactive aggression (*β* = 0.09, *p* > 0.05).

## 4 Discussion

This study investigated the longitudinal relationship between executive dysfunction and adolescent proactive and reactive aggression and explored the potential mechanisms and sex differences in the relationship between executive dysfunction and aggressive behavior based on the mediating variable of impulsivity.

### 4.1 The impact of executive dysfunction on reactive and proactive aggression

The results of this study are consistent with those of previous research (23), indicating that prior executive dysfunction significantly and positively predicts both reactive and proactive aggression among Chinese adolescents, supporting hypotheses 1a and 1b. Executive dysfunction has long been associated with various forms of aggression, including proactive and reactive aggression (55). Specifically, reactive aggression, often driven by frustration and emotional responses, has been shown to have a stronger association with executive dysfunction than proactive aggression. Hu et al. (56) highlighted that adolescents with executive dysfunction are more prone to reactive aggression, resorting to retaliatory behavior after experiencing negative emotions. This is consistent with findings from Tonnaer et al. (55), which suggest that impairments in response inhibition—a key component of executive function—are stronger predictors of reactive aggressive behavior than other executive capacities.Proactive aggression, in contrast, involves planned and goal-directed behavior that serves a strategic purpose (12). While it requires some degree of planning and self-regulation, previous research has found that deficits in these areas of executive function, such as planning and organizational abilities, may impair an individual’s capacity for proactive aggression (22). In this study, however, executive dysfunction was found to predict both reactive and proactive aggression, suggesting a more generalized impact on aggressive behavior, beyond the differences between reactive and proactive subtypes.

Interestingly, contrasting with our results, Hecht and Latzman (57) found that proactive aggression is associated with higher levels of working memory, which is a core component of executive function. The participants in this study were middle school students, while the participants in the study by Hecht and Latzman (57) were college students. Such a difference in age may be one reason for the inconsistent results. Similar to the results of this study, Jakubovic and Drabick (58) found in their investigation that lower working memory was associated with greater proactive aggression in adolescents. Working memory undergoes reorganization in brain-processing functions at the onset of puberty, typically around adolescence (59). With age, the development of working memory shifts from the activation of visuo-spatial or motor networks to the activation of executive networks (60). This change may mean that there are differences in the mechanisms underlying proactive aggression between adolescents and adults (61). In addition, response inhibition is a major predictor of aggressive behavior (62). The inhibition of executive function may not only affect the impulsive and aggressive behavior exhibited in response to provocation or conflict in reactive aggression (21, 57) but also influence individuals’ over-positive evaluation of the consequences of aggressive behavior in proactive aggression. Therefore, executive dysfunction can also positively predict proactive aggression in adolescents.

### 4.2 Differences in the predictive levels of impulsivity for reactive and proactive aggression

The link between impulsivity and aggressive behavior has been well-established in numerous studies (63–65), and other research has found that impulsivity may have different relationships with different subtypes of aggression (66). This study further verified this point through a comparison of correlation coefficients. The results showed that impulsivity had a stronger predictive effect on reactive aggression than on proactive aggression. This is consistent with findings of previous research (29, 30) indicating that impulsivity has a greater influence on individuals’ impulsive behavior when they are faced with provocation, while its predictive effect on planned proactive aggression is relatively weaker.

Reactive aggression and proactive aggression, as two subtypes of aggression, share a significant amount of conceptual and cognitive overlap, and Curtis et al. (31) argued that there is no significant difference between these two types of aggression. However, the results of this study and other previous studies show that the effects of impulsivity on reactive and proactive aggression are different. In the case of provocation, impulsivity is a key predictor of high levels of retaliation (reactive aggression), and its role is more important than those of other forms of self-control (67). Greater levels of impulsivity are also associated with increased reactive aggression, both at the initial level and in longitudinal processes over time, with a close link present between impulsivity and reactive aggression (68). Proactive aggression, as a planned and unemotional form of aggression, is more influenced by cold-hearted traits and self-aggression associations than impulsivity (29, 69).

### 4.3 The moderating role of sex in the relationship between impulsivity and aggressive behavior

The findings of this study are consistent with those of Dinić and Wertag (39), indicating that impulsivity has a stronger predictive effect on proactive aggression in male adolescents than female ones. This finding confirms the significant moderating role of sex in the relationship between impulsivity and proactive aggressive behavior, supporting hypothesis 3a. The I^3^ model of aggression suggests that aggressive behavior arises from the interaction of personality traits like impulsivity, self-regulatory capacities like executive function, and external environmental factors (26). This theory provides a framework for understanding differences in the expression of aggressive behavior between sexs. In social environments, individuals of different sexs not only have physiological differences but also experience different social lives.

Proactive aggression, as a planned and unemotional behavior, is influenced by individuals’ sensitivity to rewards and punishments (12), and this sensitivity is significantly different between sexs. Bresin (66) pointed out that males are more sensitive to seeking stimulation and rewards, while females are more sensitive to punishment. In addition to the physiological influences of serotonin and testosterone, this difference can also be explained in the I^3^ theory as an interaction between sex and the external environment. Due to the encouragement of direct and competitive behavior in the socialization process, males show greater sensitivity to impulsivity (70), and their degree of impulsivity is generally higher than that of females (71). These physiological and socialization tendencies may make males more likely to resort to aggressive behavior as a response when faced with impulsive challenges. In contrast, females’ impulsivity may be inhibited by socialization factors in the context of proactive aggression (44). Societal expectations of female behavior, such as maintaining harmonious interpersonal relationships, may reduce their impulsive expression of aggressive behavior. This socialization expectation constitutes an important factor in the external environment, demonstrating the important influence of social factors of sex on aggressive behavior.

Unlike proactive aggression, simple slope tests revealed that impulsivity is a strong predictor of reactive aggression for both boys and girls, with a more significant effect observed in boys. This finding is supported by Vaughan et al. (68), who demonstrated that the relationship between impulsivity and reactive aggression symptoms was notable in both sexes. However, the study indicated that the predictive power of impulsivity for reactive aggression is greater in males compared to females, which reflects the distinct characteristics of aggression subtypes across genders (72). Additionally, it highlights the complex interplay between cognitive processes and behavior (19).

This pattern aligns with findings from Cano-Lozano et al. (73), which indicated that boys are more likely to exhibit reactive violence toward fathers in response to parental victimization, while girls demonstrated more reactive violence toward both parents in various victimization contexts. Furthermore, Navas-Martínez and Cano-Lozano (74) emphasized that girls in the generalist profile tend to engage in psychological and control/domain violence toward mothers, suggesting that their aggression is often reactive to familial dynamics. In contrast, boys, who are found to exercise more physical violence, may be socialized to display more overtly aggressive behaviors. This divergence may be rooted in the different socialization processes that influence how boys and girls learn to respond to conflict, thus reinforcing the notion that impulsivity manifests differently across genders.

Taken together, the I^3^ model offers a multidimensional framework that allows for a deeper understanding of how sex, as part of the external environment, influences individuals’ impulsivity and aggressive behavior (26). Sex differences are not only reflected at the physiological level but are also deeply rooted in the socio-cultural context and socialization process. By utilizing this model, we can consider personality traits, self-regulatory capacities, external environmental factors, and their interactions to more comprehensively analyze and predict the occurrence of aggressive behavior. This approach can also inform the development of effective intervention strategies to reduce aggressive behavior.

### 4.4 Practical implications

In terms of practical implications, the findings of this study suggest that we should pay close attention to the impact of executive dysfunction, impulsive personality traits, and sex on adolescent aggressive behavior and give them due consideration when developing prevention strategies.

Research has shown that executive dysfunction, especially that related to behavioral disinhibition, is significantly prevalent among adolescents with antisocial behavior (75). These adolescents are more likely to engage in impulsive and risky behaviors (28), including substance abuse, gambling, and aggression (76). Fortunately, executive function can be improved through training and intervention (77), and adolescence is a critical period for prevention and intervention of executive dysfunction (78). Schools and communities can implement effective intervention measures during this period, with a particular focus on the role of executive dysfunction and impulsivity in adolescent aggressive behavior, such as fostering students’ socio-emotional learning skills, teaching them how to recognize and regulate emotions, build positive relationships, manage conflict, and make responsible decisions (79). Clinicians can also design targeted interventions, such as cognitive–behavioral therapy, based on a deep understanding of the mediating role of impulsivity in the relationship between executive dysfunction and aggressive behavior. This will effectively help adolescents identify external cues, manage their emotions, learn more adaptive behaviors, and reduce impulsive aggression (7).

In addition, sex-differentiated intervention strategies cannot be ignored. The results of this study revealed a significant moderating effect of sex on the relationship between impulsivity and aggression. Impulsivity significantly mediates the relationship between executive dysfunction and both proactive and reactive aggression in male groups. However, impulsivity does not have a significant mediating effect on proactive aggression in females, which suggests that there may be social factors beyond impulsivity that influence proactive aggression in females. Modern intervention methods emphasize the importance of sex-sensitive interventions (39). When developing intervention strategies, we must recognize the impact of sex differences on adolescent aggressive behavior and design differentiated approaches accordingly.

For male adolescents, the strong predictive role of impulsivity in aggressive behavior suggests that interventions should focus on impulse control and emotion regulation. Impulse control training can teach them self-regulation strategies during emotional arousal (42). For example, anger control training can reduce impulsivity and aggression through relaxation techniques like deep breathing and problem-solving strategies. Lei et al. (80) found that self-control strategies effectively reduced aggressive behavior among suspended adolescents. Additionally, emotion regulation training can help male adolescents manage negative emotions, such as anger, and respond more appropriately in emotionally charged situations. In contrast, female adolescents’ proactive aggression may be more influenced by social role expectations, so interventions should emphasize social-emotional learning. This can help develop empathy and communication skills, enabling non-violent conflict resolution (44). Shechtman (81) showed that short-term multidimensional interventions, incorporating bibliotherapy, effectively reduced aggression and improved emotional regulation and social skills. These interventions can promote healthier coping strategies in social contexts for female adolescents.

### 4.5 Limitations

Although this study provides an understanding of the role of executive dysfunction, impulsivity, and sex in adolescent aggressive behavior, there are still some limitations. First, the study mainly focuses on individual factors, such as impulsivity and sex, while environmental variables—specifically, parental influence and peer relationships—remain unexamined. Research indicates that parental styles, such as authoritarian or permissive approaches, can significantly affect adolescents’ behavioral outcomes (82). Similarly, peer relationships can either mitigate or exacerbate aggressive behaviors, depending on the nature of those interactions (83, 84). By failing to account for these critical environmental factors, the current study may miss key contextual influences that shape adolescent behavior.Furthermore, acknowledging these unmeasured variables is essential, as they may serve as potential confounders that could distort the study’s findings. For instance, high levels of impulsivity may be exacerbated by negative peer influences or inadequate parental support, which in turn could contribute to increased aggression. Future research should incorporate these environmental factors to provide a more nuanced understanding of the dynamics at play. Second, the reliance on self-reported data in this study introduces potential subjective bias, limiting the objectivity and accuracy of the results. Future research could enhance reliability by triangulating data from multiple sources, such as teacher ratings or peer nominations (85), which would provide a more comprehensive view of adolescent behavior. Finally, the one-year interval between measurements constrains the ability to fully explore the long-term predictive effects of executive dysfunction on aggressive behavior. Employing more complex statistical methods, such as latent growth modeling, could elucidate the long-term dynamics between these variables (68), ultimately offering stronger evidence for long-term interventions in adolescent mental health.By overcoming these limitations, future research will be able to provide deeper insights and offer more precise guidance for adolescent mental health and behavioral development.

## 5 Conclusions

The following conclusions can be drawn from this study: (1) executive dysfunction significantly and positively predicts proactive and reactive aggression; (2) impulsivity partially mediates the relationship between executive dysfunction and proactive and reactive aggression; (3) in proactive aggression, impulsivity displays a significant predictive effect only among male adolescents; and (4), in reactive aggression, impulsivity has a significant predictive effect on both male and female adolescents, although the effect is more pronounced in males.

## Data Availability

The original contributions presented in the study are publicly available. This data can be found here: OSF storage, https://osf.io/3emwr/.
